# The PRO-AGE study: an international randomised controlled study of health risk appraisal for older persons based in general practice

**DOI:** 10.1186/1471-2288-7-2

**Published:** 2007-01-11

**Authors:** Andreas E Stuck, Kalpa Kharicha, Ulrike Dapp, Jennifer Anders, Wolfgang von Renteln-Kruse, Hans Peter Meier-Baumgartner, Steve Iliffe, Danielle Harari, Martin D Bachmann, Matthias Egger, Gerhard Gillmann, John C Beck, Cameron G Swift

**Affiliations:** 1 University Department of Geriatrics, Spital Bern-Ziegler, Morillonstrasse 75-91, CH-3001 Bern, Switzerland; 2 Division of Geriatrics, Department of General Internal Medicine, Insel University Hospital, CH-3010 Bern, Switzerland; 3 Department of Primary Care and Population Sciences, University College London, Hampstead Campus, Rowland Hill Street, London NW3 2PF, UK; 4 Albertinen-Haus Geriatrics Centre, University of Hamburg, Sellhopsweg 18-22, D-22459 Hamburg, Germany; 5 Department of Social and Preventive Medicine, University of Bern, Finkenhubelweg 11, CH-3012 Bern, Switzerland; 6 Department of Ageing and Health, Guys and St Thomas' NHS Foundation Trust, 9th Floor North Wing, St Thomas' Hospital, Lambeth Palace Road, London SE1 7EH, UK; 7 School of Medicine, University of California School of Medicine, 10833 Le Conte Ave. 32-144, Los Angeles CA-90024-1687, USA; 8 Department of Health Care of the Elderly, Kings College London, Clinical Age Research Unit, King's College Hospital, Bessemer Road, London SE5 9PJ, UK

## Abstract

**Background:**

This paper describes the study protocol, the recruitment, and base-line data for evaluating the success of randomisation of the PRO-AGE (PRevention in Older people – Assessment in GEneralists' practices) project.

**Methods/Design:**

A group of general practitioners (GPs) in London (U.K.), Hamburg (Germany) and Solothurn (Switzerland) were trained in risk identification, health promotion, and prevention in older people. Their non-disabled older patients were invited to participate in a randomised controlled study. Participants allocated to the intervention group were offered the Health Risk Appraisal for Older Persons (HRA-O) instrument with a site-specific method for reinforcement (London: physician reminders in electronic medical record; Hamburg: one group session or two preventive home visits; Solothurn: six-monthly preventive home visits over a two-year period). Participants allocated to the control group received usual care. At each site, an additional group of GPs did not receive the training, and their eligible patients were invited to participate in a concurrent comparison group. Primary outcomes are self-reported health behaviour and preventative care use at one-year follow-up. In Solothurn, an additional follow-up was conducted at two years. The number of older persons agreeing to participate (% of eligible persons) in the randomised controlled study was 2503 (66.0%) in London, 2580 (53.6%) in Hamburg, and 2284 (67.5%) in Solothurn. Base-line findings confirm that randomisation of participants was successful, with comparable characteristics between intervention and control groups. The number of persons (% of eligible) enrolled in the concurrent comparison group was 636 (48.8%) in London, 746 (35.7%) in Hamburg, and 1171 (63.0%) in Solothurn.

**Discussion:**

PRO-AGE is the first large-scale randomised controlled trial of health risk appraisal for older people in Europe. Its results will inform about the effects of implementing HRA-O with different methods of reinforcement.

## Background

The development and implementation of effective interventions to prevent or delay disability in older people is an important public health priority. One promising approach is based on Health Risk Appraisal (HRA). HRA interventions were originally developed in the U.S. and tested in working age adults with the aim of identifying risks and decreasing the rate of premature illness and mortality [1]. Subsequently, HRA was adapted for use in older persons, emphasising health behaviour and preventative care in this age group [2-5]. Multiple controlled trials conducted in the United States addressed short or medium term effects of HRA and showed positive effects on health behaviour and uptake of preventative care in older persons, provided HRA was combined with a system of personal reinforcement (supplemental counselling by a physician, health educator, or other health professional) [3].

Until now, the effects of HRA in older persons have not been evaluated in European settings. The effects of the intervention might be influenced by the characteristics of health care systems or cultural factors that differ between North America and Europe. The PRO-AGE (PRevention in Older people – Assessment in GEneralists' practices) project was carried out in London (U.K.), Hamburg (Germany) and Solothurn (Switzerland) to examine the effects of a HRA for Older Persons (HRA-O) [5-8]. Here we describe the study protocol of the PRO-AGE project, and report findings related to the recruitment process and the randomisation of study participants.

## Methods/Design

**Figure 1:**
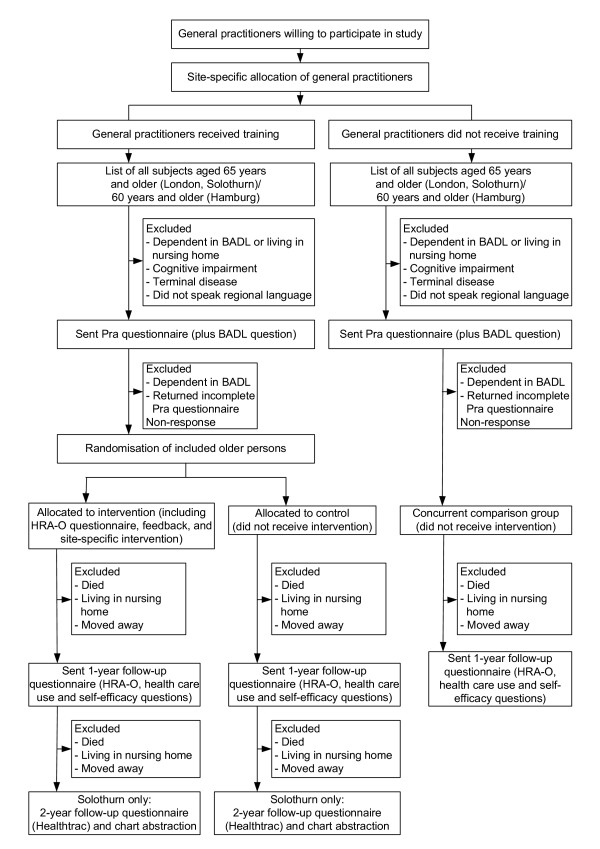
Overall design of PRO-AGE study at the three participating sites (BADL denotes basic activities of daily living; HRA-O denotes Health Risk Appraisal for Older Persons; Pra denotes Probability of repeated admissions)

**Figure 2:**
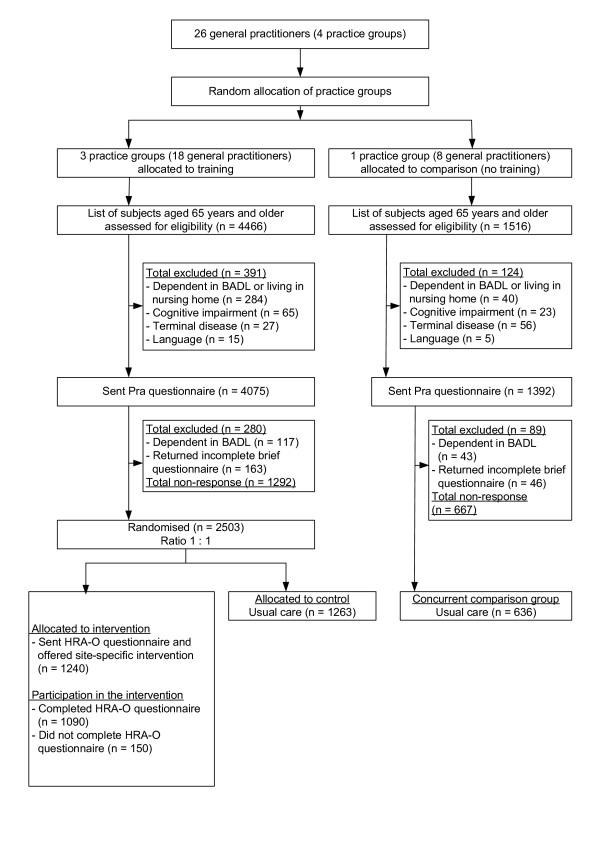
Recruitment and allocation of older persons in London (U.K.) (BADL denotes basic activities of daily living; HRA-O denotes Health Risk Appraisal for Older Persons; Pra denotes Probability of repeated admissions)

**Figure 3:**
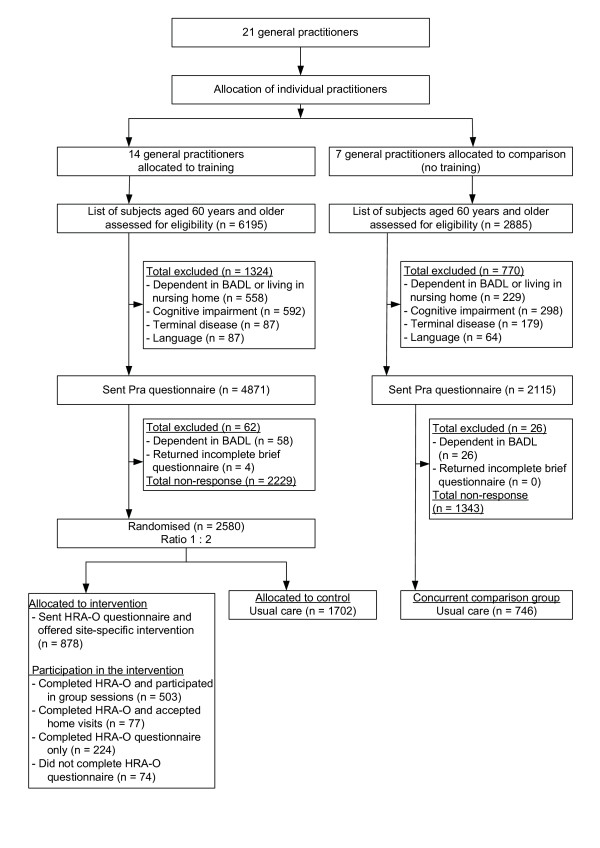
Recruitment and allocation of older persons in Hamburg (Germany) (BADL denotes basic activities of daily living; HRA-O denotes Health Risk Appraisal for Older Persons; Pra denotes Probability of repeated admissions)

**Figure 4:**
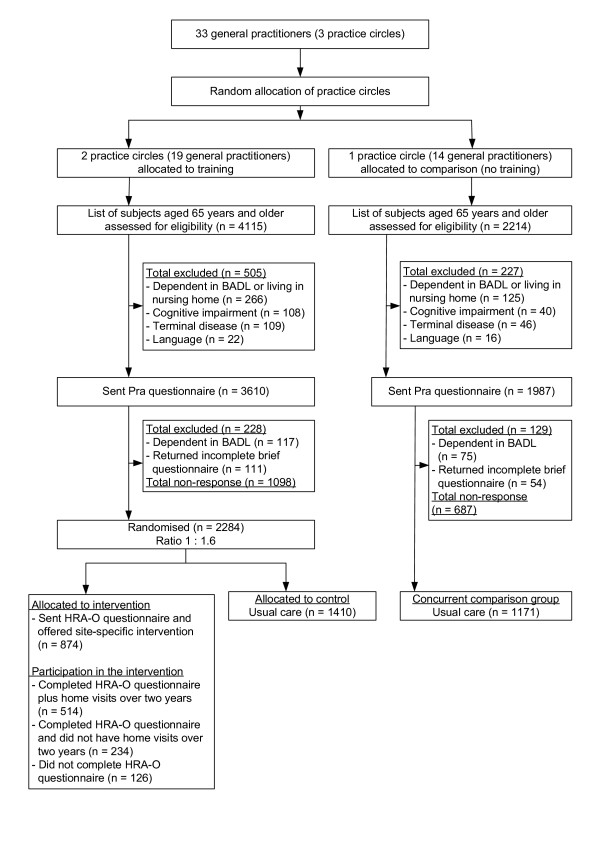
Recruitment and allocation of older persons in Solothurn (Switzerland) (BADL denotes basic activities of daily living; HRA-O denotes Health Risk Appraisal for Older Persons; Pra denotes Probability of repeated admissions)

The PRO-AGE project took place in three locations: the cities of London (U.K.) and Hamburg (Germany) and selected rural areas of the canton of Solothurn (Switzerland). The overall study protocol is summarised in Figure 1, and the recruitment process is given for each site separately (Figures 2, 3, 4). The study was approved by the regional ethical committees (see Acknowledgment section for details).

### Recruitment of general practitioners (GPs)

In London, the criteria for inclusion of general practitioners (GPs) were a known interest in primary care for older people, routine use of computerised practice information systems, and practice location in suburban London. Overall, 4 group practices with a total of 26 GPs were recruited (Figure 2). The independent study centre randomly allocated three group practices (18 GPs) to training in risk identification and preventative geriatrics, and one group practice (8 GPs) to the concurrent comparison group.

In Hamburg, GPs registered in the entire metropolitan area were invited to participate via the newsletter of their regional GP association (BDA-Landesverband Hamburg). Overall, 21 GPs (organised in solo practices) agreed to participate (Figure 3) and the Hamburg study centre formed seven groups of three GPs matched for physician age, gender, and qualification (family practice versus internal medicine). In each group of three practitioners, the Hamburg study centre allocated two GPs to training and one GP to the concurrent comparison cohort.

In Solothurn, the cantonal authority selected three mainly rural primary care service areas. The Solothurn study centre obtained agreement to participate from all 33 GPs practising in these pre-selected areas (Figure 4). In each project area, these GPs (mostly organised in single-handed or small group practices) collaborated in practice circles consisting of 8 to 14 GPs each. Each practice circle had monthly meetings to organise emergency care in the region, to exchange practice policy information, and for continuing education. The independent study centre randomly allocated two practice circles to training and those from the remaining circle to the concurrent comparison group.

### Recruitment of older persons

Participating GPs recruited older persons to the project in four steps (Figure 1). Firstly, all practices generated lists of their older patients. The age cut-off for listing patients was based on the country-specific age used for planning and delivery of services to the elderly. In London and Solothurn, an age cut-off of 65 years was selected, in Hamburg a cut-off of 60 years. In a second step, practices were asked to exclude patients meeting the following exclusion criteria: needing human assistance for performing basic activities of daily living or living in a nursing/residential home (in Germany, nursing care according to the German long-term care insurance system); cognitive impairment (i.e. possible dementia, equivalent to a Mini Mental Status score of 24 or less [9]; terminal disease; and/or inability to speak the regional language. In a third step, GPs sent an invitation letter to all individuals remaining on the list. The invitation included an information sheet describing the planned project, the Pra (Probability of repeated admissions) questionnaire [10,11], and a self-administered question on self-perceived need for human help in basic activities of daily living [12]. Patients were asked to return the completed questionnaire if they were interested in participating in the study, and to give informed consent according to the policies of the responsible institution. In London and Solothurn patients were asked to complete and return a written consent form. In Hamburg patients were informed that by returning the questionnaire they agreed to participate in the study. All patients were informed that they could withdraw from the project without negative impact on their care. In a fourth step, persons reporting a need for human assistance in performing basic activities of daily living in the questionnaire, those returning an incomplete Pra questionnaire, and those declining participation (by not returning the brief questionnaire or by returning the questionnaire with an explicit statement of refusal) were excluded from the study. The remaining persons were included in the final list of participants in the study.

### Randomisation of participants

Participating patients of GPs who had received the training were randomly allocated to intervention and control groups by the independent study centre using a computer generated allocation sequence. People living in the same household were allocated to the same group. The ratio of allocation of participants to intervention and control groups was 1:1 in the London sample and 1:2 in the Hamburg sample. In the Solothurn sample, a 1:1 randomisation ratio was used in the initial study phase, and then changed to 1:2, resulting in an average ratio of 1:1.6. Variation in randomisation rates was related to budgetary constraints (see sample size calculation), which became apparent after the project was underway. Participants allocated to the intervention group were offered HRA-O with personal reinforcement. Those allocated to the control group received usual care.

Participants of GPs who had not received additional training formed the concurrent comparison group. All patients in this group continued to receive usual care during the study period. GPs in this group received only general project information during the study period, and were offered the training after the end of the project follow-up.

Final inclusion of study participants started in November 2000 and was completed in London in October 2001, in Hamburg in September 2001, and in Solothurn in February 2002.

### The intervention

#### General goals of the interventions

The intervention evaluated in this trial consists of several components as summarised in Table 1. All components are based on the principles of risk and problem identification, achievement of favourable behaviour changes in older persons, and facilitation of preventative care use. The intervention consists of a written component (HRA-O questionnaire and feedback reports to older persons and providers) and personal patient education using multiple modalities with involvement of GPs and other health care professionals.

**Table 1:** Description of the HRA-O intervention as implemented in the PRO-AGE study

**Aspect of the intervention**	**Description**
General goals of the	To identify risks for functional decline and problems
intervention	To achieve favourable change in health-related behaviour
	To facilitate preventative care use
Training of health professionals	Use of a specially prepared manual as a basis for training of GPs and additional health professionals involved in the intervention (copy available, see additional available material)
	Initial and follow-up training of GPs and additional health professionals involved in the intervention in groups, led by one of the project physicians trained in preventative geriatric medicine
Use of HRA-O instrument	Mailing of HRA-O questionnaire to participants (copy available, see reference No. 7)
	Written individualised participant feed-back report
	Written provider HRA-O summary feed-back report
Personal reinforcement of	GP verifies presence of identified risks and problems (as described in HRA-O summary report)
HRA-O by GP	Patient discusses recommendations of participant feed-back report with their GP (opportunistically at GP-patient encounter)
	GP motivates the patient to favourably change health behaviour and use recommended preventative care (opportunistically at GP-patient encounter)
Additional site-specific	London: GP gets reminders of identified risks and problems in EMR
reinforcement	Hamburg: Participants are offered one group session by interdisciplinary team or two home visits by nurse
	Solothurn: Participants are offered six-monthly home visits by health nurses over a two-year period

HRA-O: Health Risk Appraisal for Older Persons

PRO-AGE: PRevention in Older people – Assessment in GEneralists' practices

GP: General practitioner

EMR: Electronic medical record

#### Training of health professionals

GPs (and practice nurses in London) allocated to training were trained in risk identification, health promotion and prevention in older persons. This included initial (duration about 2 hours) and follow-up (every two to three months) interactive training group sessions in risk and problem identification, and in prevention and health promotion for older persons. Training sessions were led by one of the project physicians with expertise in preventative geriatric medicine. GPs were updated on emerging evidence, health promotion and preventative messages were reinforced, difficulties encountered as part of the study raised, and problem solving strategies discussed. As a reference guide, an evidence-based training manual was developed. This manual explained the HRA-O approach, and contained current preventative care recommendations for each of the domains included in the HRA-O with relevant literature related to the management of each of the domains and references supporting the recommendations made to both participants and their physicians [see Additional file 1]. This manual was also used as a basis for training of the other health professionals involved in the intervention.

#### Use of HRA-O instrument

The intervention is based on the HRA-O instrument consisting of a self-administered HRA-O questionnaire, a personalised feed-back report to the older person and a summary feed-back report for the GP or the health educator. The development, practicability and performance of the HRA-O questionnaire are described in a separate publication [7]. Briefly, the HRA-O questionnaire contains the following sections: Administrative information (name, address, date of birth, date of completion, completion time); self-reported chronic conditions, preventative care use, medication use, signs and symptoms, self-perceived health, physical activity, nutrition, injury prevention, tobacco use, alcohol use, eyesight, hearing, depressive symptoms, self-reported memory, social network, social support, basic and instrumental activities of daily living, socio-economic information (education, occupation, living arrangement, and selected additional items at each site, such as information on state pension in London), and health measurements (weight, height, blood pressure, cholesterol). In addition, it contains additional items based on the transtheoretical model of behaviour change, as a basis for addressing the participants' readiness to change and self-perceived barriers to changing health behaviour in the feed-back statements [13]. Completed questionnaires are entered into the computer, and an algorithm-based method automatically generates summary reports for the involved health professionals, as well as personalised feed-back reports for the participants. This includes individually tailored information and recommendations based on the older persons' responses to the questionnaire, general health information on each domain addressed in the HRA-O questionnaire, and sources of further information in the community.

In the PRO-AGE study, GPs or the study centre posted the approximately 34-page HRA-O questionnaire to all individuals allocated to the intervention arm and asked them to complete the questionnaire on their own or with the support of a proxy [7]. Participants who completed the HRA-O questionnaire and their health care providers received the computer-generated personalised feed-back reports generated by the study centre at each project site.

#### Reinforcement of HRA-O

GPs (and practice nurses in London) were encouraged to reinforce the HRA-O during usual encounters with their patients. GPs were encouraged to verify problems and risks identified with the HRA-O questionnaire, and to reinforce HRA-O based recommendations by motivating patients to change their behaviour, or by facilitating preventative care uptake. In addition, participant reports encouraged patients to discuss identified risks and written recommendations with their GP if necessary.

#### Additional reinforcement in London

London adopted a specific approach for using the provider summary report. From the physician summary report, participating GPs chose to enter all or some of the HRA-O feedback into the electronic patient record (EPR) using the Read code system, (a coding system used for the classification of medical problems in EPRs in the U.K.) [14]. This choice was made by the patient's usual GP, who marked the relevant sections of the provider feedback report and passed it to a data entry clerk. The information that the patient had completed the HRA-O questionnaire and had therefore received written recommendations was added as a reminder to the EPR, and in addition individual HRA-O identified risks were incorporated as reminders to the problem list of the EPR to act as electronic prompts when the record was accessed. Finally, the whole provider summary report was scanned into the EPR as if it were a hospital letter. It was left to the discretion of both providers and patients how HRA-O identified issues were addressed, be it directly, opportunistically during unrelated consultation, or not at all. Six months after receiving the feedback report, patients in the intervention group were sent a reminder card encouraging them to follow-up the recommendations in their report and consult the practice team if necessary.

#### Additional reinforcement in Hamburg

In Hamburg, all patients allocated to the intervention group were invited to participate in a 4-hour group session at a geriatric centre or to receive two home visits by a specially trained nurse [15-18]. Group sessions were given jointly by an interdisciplinary team consisting of a geriatrician, a physical therapist, a nutritionist, and a social worker working with a structured programme focusing first on successful aging, nutrition, preventative care, lifestyle modification, physical activity, medication use, social contacts, housing and living location and emphasising patient self-efficacy and empowerment. The second part of the group session covered nutrition and physical activity in more detail. The purpose was to encourage the setting of individual goals. In the third part of the group session participants received individual written recommendations from members of the interdisciplinary team [15,16]. Older persons who preferred home visits were offered two home visits (after base-line and at six months) by a specially trained nurse who conducted an additional multidimensional assessment of nutrition, medication use, pain, mobility, cognition, vision, and hearing, prioritised recommendations and follow-up for adherence with the recommendations [17,18].

#### Additional reinforcement in Solothurn

In Solothurn, all participants who returned the HRA-O questionnaire were offered home visits by specially trained health nurses. The nurses discussed the participant feed-back on the completed HRA-O questionnaire with the older persons, answered questions from patients, and conducted a detailed interview on physical activity and medication use in all patients. If needed, they conducted additional assessments in the areas of nutrition, medication use, pain, mobility, cognition, vision, and hearing. Nurses discussed each case with a project geriatrician, and subsequently with the GP. Recommendations were prioritised, reinforced or formulated in more detail (e.g., definition of specific recommendations related to physical activity), and if new risks were identified, new recommendations were formulated. Based on the transtheoretical model of behaviour change nurses attempted to achieve behaviour change by taking into account individual readiness to change and negotiating realistic goals with the older persons. Subsequently, nurses conducted follow-up visits at 6-monthly intervals, with interim phone calls to patients as needed over a two-year period. At one year, the nurses conducted a yearly assessment home visit based on the follow-up HRA-O and on additional assessments as needed. Further funding, secured once the project was underway, enabled the duration of the intervention to be extended to two years in Solothurn. This allowed longer-term follow-up of the effects of HRA-O combined with preventive home visits.

### Data collection

Prior to randomisation, participant age and gender were recorded from practice registers. Information on base-line self-perceived health status, prior health care use, and instrumental support were derived from the Pra questionnaire [10]. In addition, for the participants in London, the Townsend score [19], a measure of social deprivation based on indicators of the older person's living location, was derived from the participants' address information.

At the one-year follow-up, surviving participants of all groups (intervention, control, and concurrent comparison group) were sent a HRA-O questionnaire with additional questions on health care use [10] and on patient self-efficacy [20]. This follow-up questionnaire included all items required for outcome analysis, as listed in Table 2. In addition, the one-year follow-up questionnaire was used for obtaining information on socioeconomic information and self-reported chronic conditions among participants in the control and concurrent comparison groups. No reminders were sent to persons not returning this questionnaire. To reduce the amount of missing information on preventative care in the London sample, practices were asked to review patient medical records for information on preventative care use (vaccination coverage, blood glucose and cholesterol measurement, colon cancer screening) for patients who had returned the 1-year follow-up questionnaire but had incomplete information on some items of preventative care (n = 44 intervention group, n = 33 control group, n = 20 concurrent comparison group).

**Table 2:** List of primary outcomes used in the PRO-AGE study at the follow-up: health behaviour and preventative care use

**Domain**	**One-year outcome analysis (all sites)**	**Two-year outcome analysis (Solothurn only)**
**Health behaviour**		
-Accident prevention	Driving without using seat belt [22]	Driving without using seat belt [2]
-Alcohol use	Possible hazardous or harmful alcohol use: based on age-and gender-specific limits of quantity and frequency of use [23]	Possible hazardous alcohol use: ≥ 2 drinks/day [2]
-Nutrition intake	Consumption of >2 high-fat food items per day [24]	Daily consumption of high-fat food items [2]
	Consumption of <5 fruit/fibre items per day [24]	Less than daily consumption of fruit and fibre items [2]
-Physical activity	Moderate or strenuous activity < 5 times/week [25]	Physical activity < 5 times/week [2]
-Tobacco use	Current tobacco use [5]	Current tobacco use [2]

**Preventative care use [21]**		
-Blood pressure	Self-report: blood pressure control in previous year	Medical record: blood pressure control in previous year
-Breast cancer screening	Self-report: mammography in previous 2 years	n.a.
-Cholesterol	Self-report: cholesterol measurement in previous 5 years	Medical record: cholesterol measurement in previous 5 years
-Colon cancer screening	Self-report: faecal occult blood test in previous year	Medical record: faecal occult blood test in previous year
-Dental care	Self-report: dental check in previous year	n.a.
-Diabetes screening	Self-report: blood glucose measurement in previous 3 years	Medical record: blood glucose measurement in previous 3 years
-Hearing examination	Self-report: hearing check-up in previous year	n.a.
-Influenza immunisation	Self-report: influenza vaccination in previous year	Medical record: influenza vaccination in previous year
-Pneumococcal immunisation	Self-report: pneumococcal vaccination (ever)	Medical record: pneumococcal vaccination (ever)
-Vision examination	Self-report: vision check-up in previous year	n.a.

PRO-AGE: PRevention in Older people – Assessment in GEneralists' practices

n.a. denotes not available

In Solothurn, in addition to the one-year follow-up, a two-year follow-up was conducted among persons in the randomised study (not in the concurrent comparison group). Health behaviour outcome data were collected using a two-page questionnaire based on the Healthtrac questionnaire [2]. In addition, older persons were asked for their self-perceived health status, and for need for help in basic activities of daily living. Non-responders or those who returned incomplete questionnaires, were contacted by telephone, or visited at home, and a trained research assistant, blinded for group allocation, attempted to obtain missing information. Selected preventative care measures (those mainly offered by GPs, and not by specialists, in the Swiss health care system) and selected health measurements (most recent values of blood based measurement, serum cholesterol, and serum glucose) were obtained from a review of primary care medical records. This abstraction of medical records was conducted by practice assistants based on an alphabetised list of participating patients blinded for group allocation.

At all sites, survival status and move into permanent long term care among non-responders at follow-up was obtained from GPs, and if available from registers in participating regions. Move into permanent long-term care was defined as living in a nursing home in London and Solothurn, and in Hamburg as requiring nursing care according to the German long-term care insurance system.

At the end of the follow-up period (London and Hamburg, at one-year follow-up, Solothurn at two-year-follow-up), participating GPs and other health personnel involved in the HRA-O intervention, received a structured, uniform questionnaire for written feed-back on the project.

### Sample size/power calculation

We calculated the required sample size for detecting, at a two-sided significance level of 0.05 and with a power of 80%, a postulated 30% difference in positive health risk behaviour or preventative care use in the intervention group as compared to controls at the one-year follow-up. For this calculation, a prevalence of positive behaviour or preventative care use among controls of 20% was assumed, based on pilot data in a comparable sample of community-dwelling older persons [6]. Based on a 1:1 randomisation ratio and a predicted drop-out rate at one year of 20%, the required sample size was 1000 persons per group. In Hamburg and Solothurn, due to limitations in resources available for offering the intervention (group sessions, home visits), a randomisation rate of 1:2 was preferable. Based on a 1:2 randomisation rate, and on the same drop out rate, the required sample size was 763 persons in the intervention group and 1525 persons in the control group.

### Statistical analyses

All analyses follow an a priori analytic plan. The primary outcomes are health behaviour and preventative care use, as listed in Table 2[2,5,21-25]. Secondary outcomes include mortality, need for long-term care (living in nursing home, or designated as requiring long-term care according to the German long-term care insurance system), and self-reported information on health status (vision, hearing, self-perceived health status, pain, depressive symptoms, falls history, and functional status).

To conduct planned sensitivity and subgroup analyses of primary outcomes, two composite scores will be created: one for adherence to positive health behaviour, and one for use of recommended preventative care. The percentage of health behaviour domains (Table 2) for which the patient showed a positive level of performance, and the percentage of recommended preventative care measures (Table 2) the patient had used will be determined. We will impute composite scores for individuals in whom ≤ 20% of the required outcome data were missing. Imputation will be conducted as follows: based on available baseline variables multiple linear regression models (using the complete base-line information and household status) will be derived in the subset of persons with complete data on outcomes, and subsequently, these models will be used to impute composite score estimates in persons with missing information.

Outcome analyses of the randomised controlled study will be conducted for each site separately in keeping with the inter-site variations of reinforcement methods. The main analyses will compare primary and secondary outcomes between intervention and control groups at follow-up. In these analyses, the fact that participants living in the same household were allocated to the same group will be taken into account by adding the information on household membership to generalised estimating equations (GEE) models [26,27]. Subgroup analyses will be used to evaluate whether the intervention effects differ between predefined subgroups of the population. Firstly, outcomes will be compared between older persons at high risk (Pra > 0.28) and those at low risk (Pra ≤ 0.28) for hospital use [10,11], based on the hypothesis that this type of intervention might have greater positive effects in persons at low risk [28]. Second, for the randomised study in Hamburg and Solothurn, an additional analysis will be carried out to evaluate the independent effects of the site-specific reinforcement on primary outcomes: multivariate regression analyses will be conducted using the composite scores as outcome variables, and participation status in the reinforcement as an independent variable, controlling for intervention allocation status and available participant base-line characteristics. Finally, sensitivity analyses will be conducted to evaluate for potential bias resulting from missing outcome data. First, all analyses will be repeated by adjusting GEE models for base-line age, gender, self-perceived health and number of physician visits reported. Second, primary outcome analyses will be repeated by using with composite scores of health behaviour and use of preventative care which will be derived from imputed outcome estimates in persons with partially missing information.

We will also evaluate changes in preventative care use, health behaviour, and health status between baseline and the one-year follow-up among persons in the intervention group. This analysis will be possible because persons in the intervention group received the HRA-O questionnaire both at base-line and at one-year follow-up. This analysis will adjust for household membership and base-line variables (age, gender, self-perceived health status, health care use prior to base-line). These pre-post analyses will be restricted to the intervention group since primary outcomes were not measured in the control group at baseline.

Outcome analysis of data from the concurrent comparison group will include a comparison of primary and secondary outcomes between the intervention group and the concurrent comparison group. The primary analysis will be a comparison of primary outcomes adjusted for base-line factors.

Categorical and binary outcome data will be analysed using chi-square tests; continuous outcome data will be compared using t-tests if normally distributed, Mann-Whitney U test if skewed. Number of physician visits will be analysed with ordered logistic regression models. To allow for within-household clustering, Generalised Estimating Equations (GEE), assuming an exchangeable correlation structure, will be used to analyse all outcomes [27]. Data will be analysed using the SAS programme [29].

### Recruitment and inclusion of study participants

Figures 2 to 4 depict the flow of study participants in London, Hamburg, and Solothurn. As shown, the percentage of participating older persons varied according to study site. Among patients of trained physicians, the numbers of older persons (% of eligible) agreeing to participate were 2503 (66.0%) in London, 2580 (53.6%) in Hamburg, and 2284 (67.5%) in Solothurn. In the concurrent comparison group, the number of persons (% of eligible) agreeing to participate was 636 (48.8%) in London, 746 (35.7%) in Hamburg, and 1171 (63.0%) in Solothurn.

Table 3 lists the results of the analyses comparing age and gender of persons agreeing to participate with eligible patients who declined. This analysis is based on multivariate logistic regression analyses. In London those who declined to participate were on average about one year older than participants. A similar comparison of the recruitment process of the Hamburg randomised study did not reveal statistically significant differences. The interpretability of the Hamburg data is limited because demographic information was not available for a proportion of non-participants due to state-mandated data protection issues. The comparison between participants and non-participants of the Solothurn randomised study revealed a gender difference, with men declining participation more often as compared to women. The comparison between participants and non-participants of the Solothurn concurrent comparison group revealed the same age difference as found in London, with non-responders being approximately one year older as compared to responders.

**Table 3:** Comparison of participants and non-participants in the PRO-AGE study at the three study sites

	**London (U.K.)**			Hamburg (Germany) *			**Solothurn (Switzerland)**		
	**Participants**	**Non-participants**	**P-value**	**Participants**	**Non-participants**	**P-value**	**Participants**	**Non-participants**	**P-value**

**Randomised study**									

Age (years)	74.6 ± 6.3 (2503)	75.7 ± 7.3 (1292)	<.0001	71.8 ± 7.6 (2580)	71.3 ± 8.1 (1234)	0.10	74.5 ± 6.0 (2284)	74.8 ± 6.8 (1098)	0.68
Female gender	54.9% (1375/2503)	58.3% (753/1292)	0.12	62.7% (1617/2580)	62.1% (821/1322)	0.70	56.6% (1293/2284)	52.7% (579/1098)	0.03

**Concurrent comparison group**									

Age (years)	74.9 ± 6.5 (636)	75.9 ± 7.5 (667)	0.01	72.0 ± 8.2 (746)	71.3 ± 8.5 (631)	0.31	74.2 ± 6.0 (1171)	75.1 ± 6.8 (687)	0.01
Female gender	57.6% (366/636)	57.4% (383/667)	0.63	68.1% (508/746)	63.7% (690/1084)	0.001	57.0% (667/1171)	60.0% (412/687)	0.32

PRO-AGE: PRevention in Older people – Assessment in GEneralists' practices

Values are percentages (nominator/denominator) or means ± standard deviations (denominator).

P-values based on multivariable logistic regression models including age and gender.

* In Hamburg, information on age and gender was in part missing amongst non-participants due to state-mandated data protection regulations.

### Comparison between groups of participants

Table 4 describes the available base-line characteristics of the study participants according to study sites and group allocation. This is to evaluate the success of randomisation. Within each site, prevalence rates were similar between intervention and control groups. In contrast, there were some relatively minor, but statistically significant differences between persons of the concurrent comparison group and persons of the intervention group (Table 4). Information on education, living alone status, and chronic conditions was derived from baseline HRA-O questionnaire (intervention groups: administered at the start of the project after randomisation; control and concurrent comparison groups: administered at the one-year follow-up). Missing values are due to non-response (all groups) or attrition (death, move away, and move to nursing home) in the control and concurrent comparison groups.

**Table 4:** Self-reported baseline characteristics of the PRO-AGE study participants, according to site and group assignment

**Baseline characteristics**	**London (U.K.)**			**Hamburg (Germany)**			**Solothurn (Switzerland)**		
	**Intervention group**	**Control group**	**Concurrent comparison group**	**Intervention group**	**Control group**	**Concurrent comparison group**	**Intervention group**	**Control group**	**Concurrent comparison group**

Age (years)	74.7 ± 6.4 (1240)	74.4 ± 6.2 (1263)	74.9 ± 6.5 (636)	71.9 ± 7.7 (878)	71.8 ± 7.6 (1702)	72.0 ± 8.2 (746)	74.5 ± 5.8 (874)	74.5 ± 6.1 (1410)	74.2 ± 6.0 (1171)
Female gender	55.3% (686/1240)	54.6% (689/1263)	57.5% (366/636)	61.5% (540/878)	63.3% (1077/1702)	68.2%* (509/746)	56.9% (497/874)	56.5% (796/1410)	57.0% (667/1171)
Fair/poor self-perceived health	24.5% (304/1240)	27.2% (343/1263)	27.4% (174/636)	38.6% (339/878)	38.5 (656/1702)	39.5% (295/746)	19.9% (174/874)	24.8% (349/1410)	23.6% (276/1171)
≥1 hospital admission over past 12 months	14.0% (173/1240)	14.6%) (185/1263)	14.2% (90/636)	21.2% (186/878)	21.2% (360/1702)	22.5% (168/746)	19.9% (174/874)	18.5% (261/1410)	17.7% (207/1171)
> 6 doctor visits over past 12 months	19.0% (236/1240)	24.3% (307/1263)	23.4% (149/636)	49.9% (438/878)	49.9% (849/1702)	44.0%* (328/746)	24.0% (210/874)	24.3% (343/1410)	28.7%* (336/1171)
No available caregiver if needed	17.6% (218/1240)	14.8% (187/1263)	19.2% (122/636)	17.8% (156/878)	18.9% (322/1702)	20.0% (149/746)	9.8% (86/874)	11.6% (163/1410)	8.8% (103/1171)
Pra score	0.27 ± 0.11	0.27 ± 0.11	0.27 ± 0.11	0.30 ± 0.11	0.30 ± 0.12	0.29 ± 0.11	0.29 ± 0.104	0.29 ± 0.11	0.30 ± 0.11
Low level of education	59.8% (666/1113)	62.5% (654/1046)	71.9%* (343/477)	18.8% (142/756)	23.5% (300/1277)	25.0%* (135/539)	47.7% (335/702)	43.2% (398/921)	41.9%* (337/804)
Living alone	33.6% (375/1116)	30.9% (324/1047))	36.4% (174/478)	35.0% (279/798)	37.4% (504/1349)	36.9% (206/559)	31.4% (234/745)	30.6% (299/976)	27.1% (229/845)
Three or more self-reported chronic conditions	33.3% (365/1095)	31.2% (324/1039)	39.0%* (183/469)	52.3% (399/763)	53.3% (691/1296)	52.3% (287/549)	39.5% (285/722)	39.6% (364/920)	42.6% (344/808)
Townsend score	1.1 ± 3.0 (1197)	1.0 ± 3.0 (1247)	4.4 ± 1.6 (635)	n.a.	n.a.	n.a.	n.a.	n.a.	n.a.

PRO-AGE: PRevention in Older people – Assessment in GEneralists' practices

n.a. denotes not available.

Values are percentages (nominator/denominator) or means ± standard deviations (denominator).

Statistically significant differences (bivariate analyses, P < 0.05) between intervention and concurrent comparison group are marked with an asterisk.

Pra score (Probability of repeated admissions): higher scores denote higher risk for hospital admission.

Townsend score: higher scores denote higher social deprivation.

## Discussion

To our knowledge this is the first randomised study of a HRA-O intervention conducted in Europe. As shown in this paper, the PRO-AGE project was successful in recruiting the large number of older persons required to evaluate – with adequate statistical power – the effects of a HRA-O intervention on health behaviour and use of preventative care. The PRO-AGE project has additional strengths. The HRA-O intervention was implemented in three sites in different countries, with different health care systems, different languages and different cultural backgrounds. Furthermore, each site implemented an additional specific reinforcement of the HRA-O. Thus, this study can evaluate the effects of HRA-O with varying forms of reinforcement in a broad range of older persons. Finally, based on the planned risk-stratified analyses using the Pra questionnaire, it will be possible to evaluate whether effects are more positive in persons at low risk, as compared to persons at high risk, as suggested by recent research evidence [28].

The PRO-AGE project has several limitations. Firstly, one-year outcome analysis is based on self-reported information alone, and there is no systematic information from direct patient observation, no cost data, and only limited information from medical records available for this analysis. However, since we used validated instruments to obtain information on a broad range of health-related measures, the study will provide valid estimates on the effects of HRA-O on health behaviour, preventative care use, and health status in older persons.

Second, outcome data will not be available for a part of the study participants due to missing data. Due to budgetary constraints it was not feasible to implement a reminder system or use some other mechanism for obtaining outcome data from persons who did not respond or only partially responded to the one-year follow-up questionnaire. The magnitude of this limitation will depend on the percentage of missing information among study participants at the three study sites. The potential impact of missing data will be evaluated in statistical sensitivity analyses.

An additional limitation of the PRO-AGE study is the fact that the study was not powered to measure the effects of the intervention on the prevention of functional decline and nursing home admissions. However, it is appropriate, as a first step, to conduct a study for identifying effective intervention methods in the short-term, and in a second step, to plan new studies to evaluate the effects of an optimised intervention strategy over longer time periods.

A further limitation of this study is the possibility of contamination effects. Patients allocated to control groups may have benefited from the intervention because they received usual care from their GPs who had received special training and were involved in the intervention (Table 1). In anticipation of this problem, the study design includes outcome evaluations in a parallel concurrent comparison group with patients receiving care from physicians who had not received training in preventative geriatric medicine. A comparison between intervention and concurrent comparison group is expected to give an estimate of treatment effects without contamination bias, but is limited due to potential selection bias. In fact, there were some small but significant differences in base-line characteristics between participants of the intervention and concurrent comparison groups. Among other factors, this might be related to the fact that the number of clusters randomised was only 4 in London, and 3 in Solothurn. To address this limitation, the analyses comparing outcomes between intervention and concurrent comparison groups will adjust for base-line characteristics.

In conclusion, recruitment of study participants to this project was successful, and base-line findings confirm the success of the randomisation and the comparability of base-line characteristics between intervention and control groups. The a priori analytic plan which examines intervention effects takes into account the described limitations, and includes sensitivity analyses evaluating potential sources of bias. Furthermore, the database containing cross-sectional and longitudinal information on multiple health risks in older people will also be useful for refining existing instruments or methods of risk prediction in older people.

## Competing interests

The author(s) declare that they have no competing interests.

## Authors' contributions

All authors are members of the PRO-AGE project group and participated in the conceptualisation and implementation of the study. HM and UD were the administrative coordinators of PRO-AGE project, AS was the technical/scientific coordinator of the project. AS, JB, CS, and HM developed the study plan. KK, DH, SI, CS implemented the London (U.K.) trial; UD, JA, WR, HM implemented the Hamburg (Germany) trial; AS was responsible for the implementation of the study in Solothurn (Switzerland). GG, ME, MB, and AS performed the central data management and data analysis. JB was involved as senior consultant to the project, and contributed to the trial design, data analysis, and data interpretation. AS and KK developed the first version of this manuscript. All authors contributed to the present manuscript.

## Contributors and additional acknowledgment

We are grateful to the practitioners and participants involved in this study.

London: Administrative and clinical staff at the Elliott Hall Medical Centre, The Ridgeway Surgery, Paxton Green Group Practice and The Forest Hill Group Practice for their commitment to and sustained involvement in this project

Hamburg: Susann Laub (Research department at the Albertinen-Haus Hamburg): Scientific nurse for the recruitment process and data collection. Norbert Lübke (Kompetenz Centrum Geriatrie Hamburg): Geriatrician for the training of General Physicians (Quality Circle) and adaption of the evidence based training manual to the German national health care system.

Solothurn (Switzerland) and PRO-AGE scientific project coordination: Christoph Minder contributed to the original proposal and analytic plan, Stephan Born was responsible for data management, Thomas Münzer and Stefan Goetz contributed to the development and implementation of the intervention programme in Solothurn.

## Ethical approval

The ethical approval of the PRO-AGE project was from the Brent Medical Ethics Committee and King's College Hospital Research Ethics Committee (London), the Ethics Committee of the Ärztekammer Hamburg (Hamburg) and the Kantonale Ethikkommission Solothurn (EKO 0023) (Solothurn).

## Pre-publication history

The pre-publication history for this paper can be accessed here:



## Supplementary Material

Additional File 1 Guidance notes for general practitioners participating in the intervention. C.G. Swift, D. Harari, S. Iliffe, N. Lübke, T. Münzer, A.E. Stuck (Jan 26, 2001). PRO-AGE: Intervention Manual (PRevention in Older persons- Assessment in Generalists' practices). Click here for file
